# Effect of opium addiction on new and traditional cardiovascular risk factors: do duration of addiction and route of administration matter?

**DOI:** 10.1186/1476-511X-7-42

**Published:** 2008-11-03

**Authors:** Sedigheh Asgary, Nizal Sarrafzadegan, Gholam-Ali Naderi, Reza Rozbehani

**Affiliations:** 1 Basic Sciences Department, Isfahan Cardiovascular Research, Center, Applied Physiology Research Center, Isfahan University of Medical Sciences, Isfahan, Iran; 2 Isfahan Cardiovascular Research Center, Isfahan University of Medical Sciences, Isfahan, Iran; 3 Faculty of Medicine, Isfahan University of Medical Sciences, Isfahan, Iran

## Abstract

**Background:**

There is a belief among some society that opium has a number of beneficial effects on cardiovascular disease. The aim of the present investigation as a cross-sectional study was to assess this hypothesis. Several biochemical factors (Fasting blood sugar, Cholesterol, Triglyceride, LDL-Cholesterol, HDL-Cholesterol, HbA1C, CRP, Fibrinogen, Factor VII, SGOT, SGPT, Lpa, apo A and apo B were evaluated in opium-addicted men (case) against non opium-addicted men(control). Three hundred and sixty opium-addicted men were divided into three groups according to the route of administration (Orally, Vafour and Sikh-Sang) and each group was divided into four subgroups according to the duration of addiction (5 months, 1 year, 2 years and 5 years). Blood morphine concentration was measured by ELISA method.

**Results:**

The results show that morphine concentration was significantly higher in orally administration. In all routes, there was a direct correlation between blood morphine concentration and period of addiction. Regardless to the period and route of administration, the level of HbA1C, CRP, factor VII, Fibrinogen, apo B, Lpa, SGOT, and SGPT were significantly higher in the case subjects as compared with controls and HDL-Cholesterol and apo a were significantly lower in the case subjects.

**Conclusion:**

This study demonstrated the deleterious effects of opium on some traditional and new cardiovascular disease risk factors. These deleterious effects are related to the period of addiction and their levels are significantly increased after 2 years of addiction. Route of administration impresses cardiovascular risk factors and "Sikh-Sang" showed the worst effect.

## Introduction

*Papaver somniferum L*. is one of the oldest cultivated plants of mankind and the only commercial source for the narcotic analgesics morphine and codeine [1,2]. Morphine is a major component of the alkaloid-rich latex in opium poppy [3]. But the plant contains about eighty different tetrahydrobenzylisoquinoline-derived alkaloids [1] which their effect on metabolism and the endocrine system could therefore be extensive and possibly different from pure morphine [3]. There are beliefs about protective cardiovascular effects of opium in some societies or even among a few physicians. It is assumed opium can be used as an alternative treatment for some of cardiovascular risk factors specially diabetes[3].

The present study was conducted to assess the effect of opium on several cardiovascular risk factors according to the duration of addiction and route of administration. Three routes of administration were chosen, orally and two form of inhalation (Vafour and Sikh-Sang) that are common in Iran. Vafour is a special opium-smoking pipe which is used to inhale the smoke. In other route (Sikh-Sang) a stiff is heat and the opium is put on the heated-stiff by a hairpin; then the smoke is inhaled by a pipe.

## Materials and methods

Three hundred and sixty opium-addicted men were divided into three groups according to the route of administration (Orally, Vafour and Sikh-Sang) and each group was divided into four subgroups according to the duration of addiction (5 months, 1 year, 2 years and 5 years). Blood morphine concentration was measured by ELISA method using Bio-quant kit (USA). The control subjects including 360 non-opium addicted but current smokers' men. All the opium addicts were cigarette smoker too. Both case and control subjects were selected consecutively from opium addicted-men and smoker men interested to quit their addiction or smoking and referred to quit addiction and rehabilitation center from Isfahan province (center of Iran). None of case and control group had any special diseases or medications and had no past history of diabetes and/or dyslipidemia. Dyslipidemia was defined as serum Cholesterol (Ch) >240 mg/dl, high density lipoprotein cholesterol (HDL-C)<35 mg/dl, low density lipoprotein(LDLC)> 135, serum triglyceride(TG)>200 mg/dl [4]. Fasting blood sugar (FBS) >100 were excluded. Subjects were informed of the aim of the study and interviews were performed by a trained nurse. Each participant signed an informed consent and self-reported opium addiction (including the route of administration and duration of opium addiction) and smoking status. The committee for medical ethics of the Isfahan Cardiovascular Research Center approved the study protocols. This committee is a member of Office for Human Research Protections, USA.

Glycosylated hemoglobin (HbA1C) was determined by column chromatography using a commercial kit (Pars Azmon, Iran). Lipoprotein a (Lpa) and quantitative C reactive protein (CRP) were measured by turbidimetric method (Hitachi 902 autoanalyzer). FBS, Ch, HDL-C, TG, SGPT and SGOT were determined by enzymatic methods (Hitachi 902 autoanalyzer). LDL-C was estimated using the Friedewald equation [5]. Plasma fibrinogen levels and factor VII were measured by thrombin-mediated clotting method. Apo B and Apo A were quantified by using a modified commercially available immunoturbidimetric assay according to the kit instruction (DiaSys, Germany)).

### Statistical analysis

Data expressed as mean ± SD were tested by student's *t *test or ANOVA and considered significant at p < 0.05. Analyses were performed with the statistical package SPSS (version 11.5, SPSS, Inc).

## Results

There was no significant difference between opium-addicted men and control group in terms of age and the smoked cigarette/day. Also, there was no significant differences between different opium addicted groups regarding smoking. The mean of smoked cigarettes/day was respectively 15 ± 2 and 16 ± 3 in opium addicted and control group. The mean age of smokers group was 38 ± 5 years and it was 41 ± 3 years for opium addicted group.

**Figure 1:**
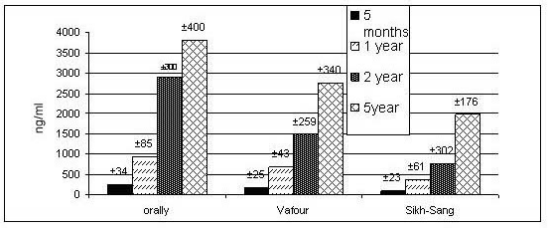
Relation between morphine concentration and duration of addiction in all the routes of administration (Orally, Vafour and Sikh-Sang).

A direct correlation was found between morphine concentration and duration of addiction in all the routes (figure 1). But, in all the durations, the higher morphine concentration was for orally route followed by "Vafour" and "Sikh-Sang" respectively (figure 1). Blood morphine level was 18 ± 4 ng/ml in smokers non addicted group.

In the opium addicted subjects, HbA1C, CRP, factor VII, Fibrinogen, Lpa, apo B, SGOT, and SGPT were significantly (p < 0.05) higher as compared with control subjects and HDL-C and apo A was significantly lower in them(table 1).

**Table 1:** Comparison of the mean of biochemical factors between addicted men and control group.

**Biochemical factors**	**Normal range**	**addict**	**Non addict**	**P value**
**FBS (mg/dl)**	70–115	79 ± 11	84 ± 15	0.46
**HbA**_1_**C (%)**	5–7.5	8.04 ± 0.91	7.12 ± 2.07	0.03
**Ch(mg/dl)**	200–239	176 ± 25	169 ± 14	0.22
**TG (mg/dl)**	≤ 220	138 ± 52	152 ± 41	0.43
**HDL-C (mg/dl)**	29–80	41 ± 5	44 ± 5	0.032
**LDL-C (mg/dl)**	≤ 130	92 ± 19	110 ± 14	0.42
**Lpa (mg/dl)**	<30	48.7 ± 3.2	25 ± 2.5	0.001
**Apo-A (mg/dl)**	110–170	117 ± 18	165 ± 32	0.01
**Apo-B (mg/dl)**	80–155	144 ± 12	124 ± 8.9	0.037
**CRP (mg/dl)**	<10	4.11 ± 0.7	3.54 ± 0.3	0.029
**SGOT (IU/L)**	5–40	39.2 ± 2.5	18.3 ± 1.8	0.02
**SGPT (IU/L)**	5–40	34.3 ± 1.8	11.4 ± 0.6	0.017
**Fibrinogen (mg/dl)**	250–400	330.2 ± 25	263 ± 17	0.042
**Factor VII (%activity)**	80–130	243.9 ± 29	123 ± 27	0.014

Data represent as mean ± SD (n = 360), p < 0.05 considered as significant

When effects of routes of administration were compared with each other (regardless to the duration of addiction), it was found that "Sikh-Sang" presented greater effect on Ch, TG, Lpa, apo B, CRP and fibrinogen (table 2). Although CRP and Fibrinogen respectively are significantly more in Vafour and orally route. Regardless to the route of administration as shown in table 3, i) FBS reduced nonsignificantly when the duration of addiction increased. ii). No significant differences were observed in Ch, TG and HDL-C when the period of addiction increased iii) Lpa, apo B, HbA1C, CRP, SGOT, SGPT, Fibrinogen, Factor VII and LDL augmented significantly when the period of addiction increased more than 2 years but apoA decreased significantly.

**Table 2:** The mean ± SD of biochemical factors in opium addicted men based on the route of addiction

**Biochemical factors**	**Vafour**	**Sikh-sang**	**Orally**
**FBS (mg/dl)**	81.3 ± 15.3	82.2 ± 13.2	74.9 ± 12.6
**HbA**_1_**C (%)**	8.9 ± 0.7	8.5 ± 0.8	8.8 ± 0.8
**Ch(mg/dl)**	172.3 ± 28.3	180.8* ± 28.6	175.5 ± 29.0
**TG (mg/dl)**	119.6 ± 69.6	160.9* ± 32.2	136.1 ± 50.6
**HDL-C (mg/dl)**	40.8 ± 4.9	38.3 ± 3.07	37.9 ± 2.7
**LDL-C (mg/dl)**	83.7 ± 38.4	107.2 ± 21.2	108.4 ± 22.4
**Lpa (mg/dl)**	47.0 ± 38.9	56.5* ± 39.1	42.1 ± 35.1
**Apo-A (mg/dl)**	118.8 ± 21.3	114.8 ± 29.3	119.9 ± 21.80
**Apo-B (mg/dl)**	124.8 ± 29.9	169.8* ± 31.3	139.6 ± 23.58
**CRP (mg/dl)**	3.7* ± 1.6	3.4* ± 1.6	2.8 ± 1.6
**SGOT (IU/L)**	26.9 ± 12.5	28.1 ± 12.1	48 ± 35.1
**SGPT (IU/L)**	28.1 ± 16.9	41.8 ± 16.3	48 ± 34.5
**Fibrinogen (mg/dl)**	275 ± 18	345* ± 37	389* ± 12
**Factor VII (%activity)**	239 ± 12	253 ± 23	257 ± 18

Data represent as mean ± SD, p < 0.05 considered as significant

**Table 3:** The mean ± SD of biochemical factors in opium addicted men based on the period of addiction

**Biochemical factors**	**5 months**	**1 year**	**2 years**	**5 years**
**FBS (mg/dl)**	80.6 ± 11.9	80.3 ± 15.96	81.9 ± 16.8	74.6 ± 9.0
**HbA**_1_**C (%)**	6.8 ± 0.9	7.2 ± 0.9	9.0* ± 0.72	9.5* ± 0.7
**Ch(mg/dl)**	174.8 ± 25.7	180.0 ± 29.0	177.5 ± 30.5	170.9 ± 27.4
**TG (mg/dl)**	124.7 ± 54.9	122.92 ± 67.3	120.8 ± 59.5	147.4 ± 48.1
**HDL-C (mg/dl)**	38.34 ± 3.2	37.3 ± 2.1	39.9 ± 3.8	38.6 ± 3.3
**LDL-C (mg/dl)**	87.2 ± 39.92	73.7 ± 45.5	105.1* ± 28.6	103.0* ± 19.2
**Lpa (mg/dl)**	35.2 ± 37.4	41.1 ± 21.1	50.9* ± 41.2	68.1* ± 37.6
**Apo-A (mg/dl)**	141.9 ± 15.11	125.8 ± 25.9	99.9* ± 22.9	100.0* ± 25.6
**Apo-B (mg/dl)**	113.4 ± 47.7	115.6 ± 41.8	152.3* ± 29.8	184.0 ± *23.6
**CRP (mg/dl)**	2.7 ± 0.2	2.7 ± 0.6	4.5* ± 0.6	5.4* ± 0.6
**SGOT (IU/L)**	18.5 ± 1.3	23.6 ± 2.4	40.2* ± 4.5	47.4* ± 5.2
**SGPT (IU/L)**	26.8 ± 2.1	29.3 ± 5.3	42.8* ± 4.8	49.2* ± 5.7
**Fibrinogen (mg/dl)**	270 ± 12	298 ± 32	408* ± 31	450* ± 12
**Factor VII (%activity)**	122 ± 14	134 ± 28	316* ± 32	342* ± 81

Data represent as mean ± SD, p < 0.05 considered as significant

## Discussion

The results show the deleterious effect of opium on some traditional and new CVD risk factors including HbA1c, HDL-C, Lpa, apo B, factor VII, fibrinogen, apo A and CRP. Lpa which was significantly more in opium addicted is an independent risk factor for premature atherosclerosis [6,7]. High levels of CRP and clotting factors that was seen in the opium addicts in the present study, suggest that use of opium increase risk for heart attack or stroke. Inflammation is recognized as a major etiologic determinant of multiple disease states including myocardial infarction, stroke, diabetes, and metabolic syndrome, and individuals with elevated levels of the inflammatory biomarkers such as CRP are at increased risk of mortality and morbidity from these conditions[8].

The presenting association between Fibrinogen and CHD beyond 10 years may imply a casual effect and also there is association between Factor VII and CHD[9]. Studies have shown that apoB and apo A are strongly predictors of myocardial infarction and future cardiovascular events[10] Meanwhile, reduction of HDL-C in opium addicts was significant. Numerous clinical and epidemiological studies have demonstrated that plasma concentrations of HDL-C and apo A are inversely linked with CVD [11]. Both HDLC and apoA have antioxidant, anti-thrombotic, anti-inflammatory properties, which may be related to their anti-atherogenic function [12-14].

Even though, FBS reduced in the opium addicted subjects but it was not significant. Furthermore, the reduction of FBS was concurrent with increase in HbA1C level then it could be concluded that reduction of FBS was not a valuable outcome because HbA1c provides an integrated measurement of blood glucose during previous 2–3 months, reflecting 120-day life span of erythrocytes [15]. Also other study is revealed that opium might decrease blood glucose temporarily [16]. These findings are also in agreement with Karam et al. [3] that HbA1C was higher in the opium addict non-insulin-dependent diabetes mellitus (NIDDM) men compared to non-opium addict NIDDM male. They have also reported that opium addiction in NIDDM subjects increased serum glucose and decreased HDL-C; and thus progress to metabolic disorders in NIDDM patients.

As it expected in all the routes, there was a direct correlation between blood morphine concentration and period of addiction. Repeated use of opiate drugs, such as morphine, leads to the development of tolerance and dependence [17] which it result in excessive drug consumption. Then it is usual that blood morphine concentration augments when the duration of addiction increase. Although, in all the duration the higher morphine concentration was reported in the route of orally but the worst effect was observed in "Sikh-Sang". This observation suggests that destructive effects of opium are related to other compounds in opium or may be the route of administration influence diverse metabolic pathways by different mechanisms. Even though, the mechanisms of these differences are not clear but different outcomes have been mentioned for various routes of opium administration. For example, it has been reported that the onset of action of opium is delayed after oral ingestion of opium (contains morphine and codeine), because opium is poorly absorbed in the stomach but well absorbed in the small intestine.

In contrast, vaporized morphine produced by smoking of opium is rapidly absorbed across the lungs into the blood stream, and within a few seconds is available at the brain. Hence the onset of action is more rapid after smoking; however, the duration of action is longer after oral ingestion [18]. This is also in agree with several reports that shown orally administrated addictive substances such as cannabinoids have a slower onset of action but due to the rapid delivery to the brain, they are more addictive when smoked than when administered by other routes [19,20]. By the way, it has been reported that morphine increases hormones such as adrenalin, noradrenalin, corticosterone, prolactin and glucagons which affect the metabolism in different ways [21]. Short- and long-term effects of morphine on the hormone secretion are different.

The reduction of TG, Ch, and LDL which was observed in the opium addicts in the present study were not significant. Furthermore, one of the main factors influencing blood lipids that are not taken into account in this study is the diet. On the other hand, nutritional habits, social status and unfavorable lifestyle behaviors could influence the blood lipid profile in a person. Usually the opium addicts have malnutrition may be due to economical problems, they prefer to save their money for buying opium instead food or loss of appetite due to the effect of opium. It is worthy to consider that this study indicate the level of cardiovascular risk factors are significantly more in opium addicted men than smoker men, while smoking is one of the important risk factor of cardiovascular disease and cancer[22-24], therefore the deleterious effects of opium addiction on cardiovascular disease are considerable.

Route of administration impresses cardiovascular risk factors and "Sikh-Sang" showed the worst effect. Effect of different temperatures on opium compounds during certain routes, effect of pipe on filtration of opium smoke in "Vafour" and effect of digestive enzymes on opium compounds during orally route should be more investigated.

## Competing interests

The authors declare that they have no competing interests.

## Authors' contributions

SA had substantial contributions to conception and design and interpretation of data and writing the manuscript. NS had substantial contributions to conception and design. GN carried out the biochemical analysis. RR had contributions to data analysis. All authors read and approved the final manuscript.
